# A low-carbohydrate ketogenic diet induces the expression of very-low-density lipoprotein receptor in liver and affects its associated metabolic abnormalities

**DOI:** 10.1038/s41538-019-0058-4

**Published:** 2019-12-02

**Authors:** Tetsuya Okuda

**Affiliations:** Bio-Design Research Group, Bioproduction Research Institute, National Institute of Advanced Industrial Science and Technology (AIST), Tsukuba, Japan

**Keywords:** Obesity, Nutrition

## Abstract

A low-carbohydrate ketogenic diet (LCKD) promotes the progression of hepatic steatosis in C57BL/6 wild-type mice, but improves the condition in leptin-deficient obese (*ob/ob*) mice. Here, we show a novel effect of LCKD associated with the conflicting effects on these mice. Gene expression microarray analyses showed that expression of the *Vldlr* gene, which encodes the very-low-density lipoprotein receptor (VLDLR), was induced in LCKD-fed *ob/ob* mice. Although the VLDLR is not normally expressed in the liver, the LCKD led to VLDLR expression in both *ob/ob* and wild-type mice. To clarify this effect on VLDL dynamics, we analyzed the lipid content of serum lipoproteins and found a marked decrease in VLDL-triglycerides only in LCKD-fed wild-type mice. Further analyses suggested that transport of triglycerides via VLDL from the liver to extrahepatic tissues was inhibited by LCKD-induced hepatic VLDLR expression, but rescued under conditions of leptin deficiency.

## Introduction

Consumption of low-carbohydrate ketogenic diets (LCKDs) has been shown to have diverse beneficial effects in promoting health and alleviating pathologic conditions. For example, LCKDs exhibit antiepileptic effects,^1^ promote weight loss in obesity,^2^ and enhance control of type 2 diabetes mellitus.^3^ LCKDs also reportedly have beneficial effects in patients with autism,^4^ cardiomyopathy,^5^ neurodegenerative diseases,^1^ and cancer.^6^ The effective and safe use of LCKDs based on scientific evidence has contributed to both health promotion and disease treatment; however, the molecular mechanisms underlying the beneficial effects of LCKDs in vivo remain largely unknown.

As a clinical trial demonstrated that restricting carbohydrate intake can reduce intrahepatic triglyceride levels,^7^ increased research attention has focused on the use of LCKDs in treating liver steatosis.^8^ However, children with epilepsy have been shown to develop liver steatosis with long-term consumption of a LCKD;^9^ thus, careful investigation of this effect is needed. A number of studies using rodent models have analyzed the role of LCKDs in the development of liver steatosis.^10–16^ Most of these studies used a common LCKD for rodents (Bio-Serv F3666),^10–13,15,16^ with subsequent development of steatosis observed in the liver of the LCKD-fed mice.^10–12,16^ However, surprisingly, we found that the F3666 diet had a dramatic preventative effect on the progression of liver steatosis in juvenile leptin-deficient obese (*ob/ob*) mice.^13^ We found that the underlying mechanism involves suppression of de novo triglyceride synthesis.^13,16^ This LCKD also suppresses de novo triglyceride synthesis in C57BL/6J (wild-type) mice that develop liver steatosis.^10–12,16^ These conflicting results suggest that the beneficial effect of the LCKD on liver steatosis in *ob/ob* mice is regulated by an as yet unknown mechanism that is non-functional in wild-type mice.

To clarify the underlying mechanism of these effects, we analyzed several molecular parameters in the liver of LCKD-fed *ob/ob* mice and found a novel effect of the LCKD in up-regulating expression of the very-low-density lipoprotein receptor (VLDLR) in the liver. Although this receptor is expressed only minimally in the liver,^17^ LCKD feeding significantly up-regulated VLDLR expression in the liver of *ob/ob* and wild-type mice. Further analyses showed that VLDL-triglyceride levels were significantly lower in the serum of LCKD-fed wild-type mice. In addition, serum activity of lipoprotein lipase (LPL), which mediates triglyceride uptake from VLDL into extrahepatic tissues,^18^ was also down-regulated. In contrast, marked release of VLDL-triglycerides was observed in LCKD-fed *ob/ob* mice, whereas LPL activity was maintained. These alterations in both strains were correlated with the conflicting liver steatosis phenotypes.

The results of our study suggest that the transport of triglycerides via VLDL from the liver to extrahepatic tissues is inhibited by LCKD-induced hepatic VLDLR up-regulation under low LPL activity, whereas this inhibition of triglyceride transport is rescued under conditions of leptin deficiency.

## Results

### Characterization of liver phenotypes in LCKD-fed mice

Wild-type and *ob/ob* mice of the inbred strain C57BL/6J were used in this study and fed the Bio-Serv F3666 LCKD. F3666 is a very-low-carbohydrate, low-protein, high-fat ketogenic diet developed to induce efficient production of ketone bodies in rodents.^19^ In *ob/ob* mutant mice fed a diet of regular chow, hyperglycemia typically develops at ~10 weeks of age.^20^ Our initial goal was to reverse this phenotype via feeding mice the LCKD and analyze the diet’s effects on tissues at the molecular level. The mice were fed the LCKD over the period 5–12 weeks of age in the dietary experiment.^13^ We found that the LCKD feeding effectively reversed the hyperglycemic phenotype in female *ob/ob* mice during this period and therefore employed these conditions in subsequent experiments.^13^ The average blood glucose levels during the experimental period were as follows: chow-fed *ob/ob* mice, 194.30 ± 43.78 mg dl^−1^; LCKD-fed *ob/ob* mice, 106.96 ± 26.41 mg dl^−1^; chow-fed wild-type mice, 154.08 ± 20.55 mg dl^−1^; and LCKD-fed wild-type mice, 114.30 ± 15.05 mg dl^−1^. Production of ketone bodies (β-hydroxybutyrate) was observed in both the *ob/ob* and wild-type mice.^13,16,21^

**Fig. 1:**
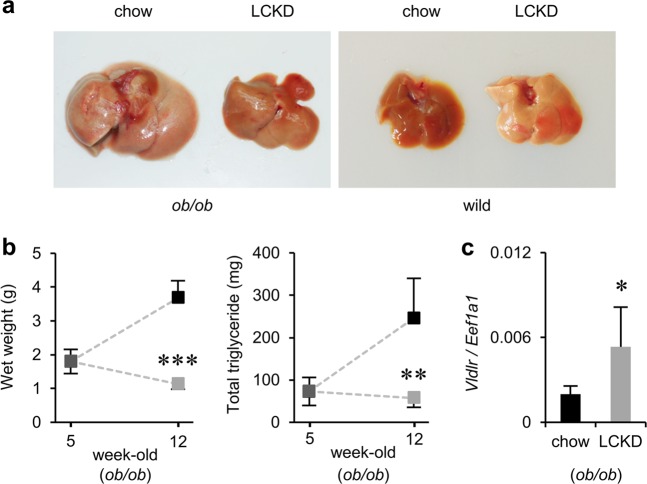
Effect of LCKD feeding on liver phenotypes. **a** Morphology of the liver after 7 weeks of feeding. **b** Wet weight (*n* = 11–13) and total triglyceride levels (*n* = 5–6) in the liver. **c** Real-time PCR analysis of expression of the *Vldlr* gene (*n* = 6). The relative expression level is shown as the ratio relative to expression of the internal standard (*Eef1a1*). Statistical significance was assessed using the two-tailed Student’s *t* test. *P < 0.05, **P < 0.01, ***P < 0.001, chow-fed vs. LCKD-fed. Black squares and bars, regular chow-fed mice; gray squares and bars, LCKD-fed mice. Mean ± SD

Figure 1a shows the morphology of the liver after 7 weeks of LCKD feeding. In *ob/ob* mice, regular chow promoted significant steatosis associated with enlargement of the liver (left panel). Liver weight and total amount of triglycerides increased by more than 2-fold compared to values prior to the start of the experiment (Fig. 1b). The LCKD is known to inhibit the progression of liver steatosis in *ob/ob* mice.^13^ Compared to chow-fed mice, the liver weight and total amount of triglycerides decreased by at least 70% in LCKD-fed mice (Fig. 1b). The average triglycerides (mg) to liver weight (g) ratios were as follows: chow-fed *ob/ob* mice, 65.75 ± 24.7 mg g^−1^; and LCKD-fed *ob/ob* mice, 48.79 ± 16.93 mg g^−1^. In contrast, the LCKD strongly promoted steatosis in wild-type mice. In LCKD-fed wild-type mice, the total amount of triglycerides in the liver has been shown to increase by more than 3-fold.^16^ Although liver weight remained unchanged in the present study, the organ became discolored as a result of excessive triglyceride accumulation (Fig. 1a, right panel). The average triglycerides (mg) to liver weight (g) ratios were as follows: chow-fed wild-type mice, 19.17 ± 5.68 mg g^−1^; and LCKD-fed wild-type mice, 63.65 ± 16.44 mg g^−1^. In both strains, body weight gain and diet intake (kcal per day) were similar in the chow- and LCKD-fed groups during the experimental period.^13,16^ Although LCKD feeding improved the steatosis associated with enlargement of the liver, LCKD-fed *ob/ob* mice became obese to the same degree as chow-fed *ob/ob* mice.^13^ The average final body weights were as follows: chow-fed *ob/ob* mice, 53.16 ± 2.45 g; LCKD-fed *ob/ob* mice, 52.47 ± 3.45 g; chow-fed wild-type mice, 19.16 ± 1.79 g; and LCKD-fed wild-type mice, 17.87 ± 2.02 g. The average caloric intake during the experimental period was as follows: chow-fed *ob/ob* mice, 18.56 ± 0.4 kcal per day; LCKD-fed *ob/ob* mice, 17.27 ± 2.43 kcal per day; chow-fed wild-type mice, 10.48 ± 0.46 kcal per day; and LCKD-fed wild-type mice, 10.41 ± 1.5 kcal per day.

### LCKD effect on hepatic gene expression

A gene expression microarray analysis was conducted to elucidate the molecular mechanism underlying the improvement of liver steatosis in LCKD-fed *ob/ob* mice. Data for all genes detected as specific signals were compared with data for mice fed the regular chow (*n* = 3) and deposited in the NCBI Gene Expression Omnibus (GEO; accession number GSE115342). The overall features of the results have been published elsewhere.^21^

A novel result observed in the present study was significant up-regulation of the *Vldlr* gene, which encodes the VLDLR. The microarray analysis revealed significant up-regulation (log^2^ ratio [LCKD/chow] of +1.78; *P* < 0.05) of *Vldlr*, which was confirmed in validation experiments using real-time PCR (Fig. 1c) with a greater number of samples (*n* = 6). No apparent effects on other factors associated with VLDL metabolism were observed in this experiment.

### Effect of the LCKD on induction of VLDLR in the liver

**Fig. 2:**
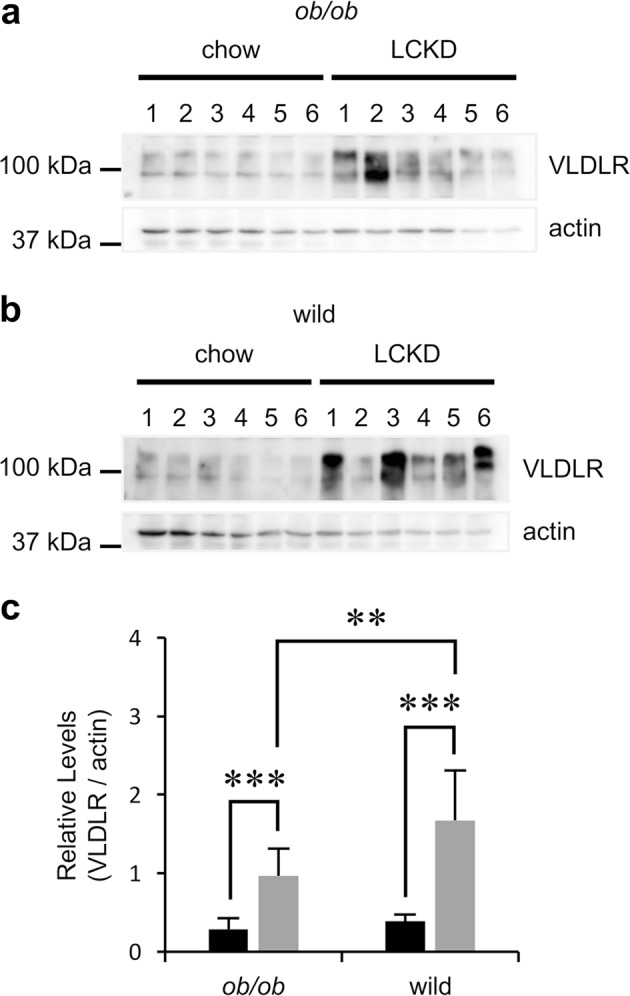
Immunoblot analysis of VLDLR in the liver. Representative images of immunoblotting of VLDLR in the liver of individual *ob/ob* **a** or wild-type **b** mice (*n* = 6). Actin is shown as an internal standard. Band intensities were analyzed as previously reported^16^ to determine relative levels (**c**, VLDLR/actin). *ob/ob*, *n* = 16–17; wild-type, *n* = 6. Statistical significance was assessed using the two-tailed Student’s *t* test. **P < 0.01, ***P < 0.001. Black squares and bars, regular chow-fed mice; gray squares and bars, LCKD-fed mice. Mean ± SD

As VLDL regulates the release of triglycerides from the liver to the blood stream,^18^ we expected that up-regulation of *Vldlr* would correlate with the liver phenotype in LCKD-fed mice. Immunoblot analysis showed that VLDLR, which is not normally expressed in the liver,^17,22^ was detected in the liver of mice fed the LCKD (Fig. 2). However, the induction of VLDLR expression was observed in both *ob/ob* (Fig. 2a) and wild-type (Fig. 2b) mice, indicating that this effect is not specific to *ob/ob* mice. Although individual differences were observed, further statistical analyses indicated that the effect was associated with the LCKD (Fig. 2c). The statistical analyses also revealed that the level of VLDLR expression in LCKD-fed wild-type mice was higher than that in *ob/ob* mice (*P* < 0.01).

### Characterization of lipid content of lipoproteins and LPL activity in serum

To clarify the effect of hepatic VLDLR on lipoprotein dynamics, we subsequently analyzed the lipid content of serum lipoproteins (Table 1). We found that levels of VLDL-triglycerides were markedly lower in LCKD-fed wild-type mice, but unchanged in LCKD-fed *ob/ob* mice. In contrast, VLDL-cholesterol levels were unchanged in LCKD-fed wild-type mice, but significantly lower in LCKD-fed *ob/ob* mice. These results indicate that the release of VLDL from the liver to the blood stream is not impaired in either LCKD-fed mouse strain; rather, only VLDL-mediated triglyceride release is specifically inhibited in LCKD-fed wild-type mice. The increase in LDL-cholesterol levels in LCKD-fed wild-type mice also supports the normal release of VLDL from the liver in these mice. In LCKD-fed *ob/ob* mice, the cholesterol content of the VLDL fraction was considerably lower than the triglyceride content, indicating that triglyceride-rich VLDL was generated in these mice. Furthermore, LDL-triglyceride levels were significantly higher in LCKD-fed *ob/ob* mice, but unchanged in LCKD-fed wild-type mice, which indicates a difference between *ob/ob* and wild-type mice in terms of conversion from VLDL-triglycerides to LDL-triglycerides. As this process is mediated by LPL,^18^ we preliminarily measured the activity of LPL in the serum and found a significant decrease only in LCKD-fed wild-type mice (Supplementary Fig. S1). In contrast, serum LPL activity was the same in LCKD- and regular chow-fed *ob/ob* mice, indicating that the LCKD-associated decrease in serum LPL activity is specific to wild-type mice. Statistical analyses also revealed that LPL activity in chow-fed *ob/ob* mice was lower than that in chow-fed wild-type mice (*P* < 0.01). However, LPL activity in LCKD-fed *ob/ob* mice was higher than that in LCKD-fed wild-type mice (*P* < 0.05).

**Table 1:** Serum lipoprotein lipid content

	Strain			
	*ob/ob*		Wild type	
Diet	Chow	LCKD	Chow	LCKD
Parameters				
Triglyceride (mmol l^−1^)				
Total	1.23 ± 0.22	1.47 ± 0.30	1.30 ± 0.20	0.18 ± 0.03**
CM	0.31 ± 0.06	0.08 ± 0.02*	0.14 ± 0.03	0.02 ± 0.005**
VLDL	0.58 ± 0.11	0.53 ± 0.14	0.97 ± 0.15	0.04 ± 0.01**
LDL	0.23 ± 0.04	0.65 ± 0.17***	0.15 ± 0.02	0.10 ± 0.01
HDL	0.08 ± 0.02	0.07 ± 0.02	0.04 ± 0.01	0.02 ± 0.002*
Cholesterol (mmol l^−1^)				
Total	4.08 ± 0.28	5.66 ± 0.18**	2.02 ± 0.11	5.08 ± 0.66**
CM	0.05 ± 0.01	0.01 ± 0.001**	0.06 ± 0.01	0.04 ± 0.02
VLDL	0.16 ± 0.03	0.09 ± 0.004***	0.44 ± 0.05	0.49 ± 0.25
LDL	0.71 ± 0.06	0.81 ± 0.04	0.31 ± 0.02	1.34 ± 0.33*
HDL	3.18 ± 0.24	4.66 ± 0.15**	1.21 ± 0.04	3.20 ± 0.12***

Values include previously reported data for *ob/ob* mice.^13^ Mean ± SD; *n* = 9 (*ob/ob*) or *n* = 6 (wild type). Statistical significance was assessed using the two-tailed Student’s *t* test, with significance defined as follows: *P < 0.05, **P < 0.01, ***P < 0.001, regular chow-fed vs. LCKD-fed mice

## Discussion

A novel effect of the LCKD on induction of VLDLR expression was found in the liver, where this receptor is not normally expressed. Previous studies reported that the peroxisome proliferator-activated receptor α agonist fenofibrate promotes not only hepatic VLDLR expression^22^ but also steatosis,^23^ which is in agreement with the phenotype of the LCKD-fed wild-type mice in the present study. The induction of hepatic VLDLR expression by fenofibrate or over-expression of VLDLR decreases triglyceride-rich VLDL levels via triglyceride clearance by the liver,^22,24^ which can promote the development of liver steatosis.

F3666 is a commonly used LCKD for rodents, and several recent studies have examined the effects of the low amounts of choline and protein in this diet.^14,15^ A lack of sufficient choline is associated with the development of steatosis in the liver,^14,15,25^ which impairs hepatic triglyceride secretion due to an absence of phosphatidylcholine synthesis.^25^ Choline supplementation can improve this pathologic condition.^14,15^ The low protein content of the F3666 diet increases the expression of several genes in the liver, but this effect is attenuated by methionine supplementation.^15^ These observations suggest that the low choline and protein content of the F3666 diet also played a role in the effects observed in our model. However, in *ob/ob* mice in our model, the F3666 diet prevented the progression of liver steatosis observed in chow-fed *ob/ob* mice (Fig. 1). Furthermore, the F3666 diet used in our study contained an abundance of choline-containing phospholipids (phosphatidylcholines and sphingomyelins, 5.8 ± 0.2 g kg^−1^), and serum levels of choline-containing phospholipids in F3666-fed mice were also significantly higher than the levels in chow-fed mice (Supplementary Fig. S2). These results indicate that choline was supplied to peripheral tissues in sufficient amounts in the mice using this study, and therefore, choline levels had a minimal effect on development of steatosis.

We also observed VLDLR expression in the liver of LCKD-fed *ob/ob* mice, in which the hyperphagic phenotype can compensate for the low protein content of the LCKD. This result indicates that the low protein content of the diet is not associated with increased VLDLR expression. As the low protein content of the F3666 diet plays an important role in the generation of ketone bodies in rodents,^19^ we conclude that the low-carbohydrate and high-fat/ketogenic properties of the LCKD induce hepatic VLDLR expression.

**Fig. 3:**
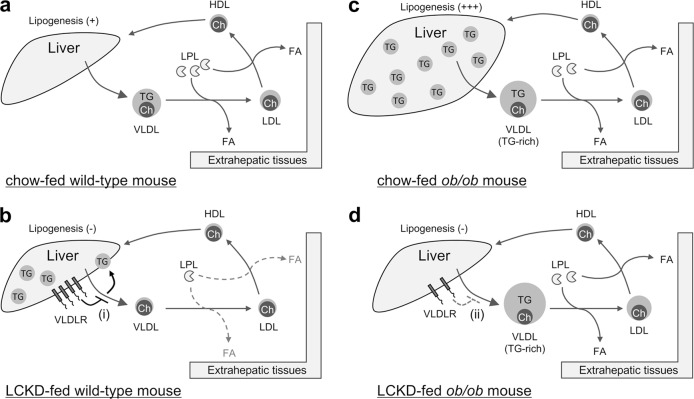
Schematic illustrations of VLDL dynamics and metabolism in LCKD-fed mice. Overview of VLDL dynamics and metabolism in chow-fed wild-type **a**, LCKD-fed wild-type mice **b**, chow-fed *ob/ob* mice **c**, and LCKD-fed *ob/ob* mice **d**. The size and number of objects are drawn to reflect the experimental results. The working hypothesis regarding the effect of hepatic VLDLR on VLDL is as follows: (i) in wild-type mice, LCKD induces hepatic VLDLR expression, which inhibits triglyceride (TG) secretion via VLDL; (ii) in *ob/ob* mice, LCKD induces hepatic VLDLR expression, whereas the VLDLR does not function for TG-rich VLDL, which is specifically generated in the *ob/ob* strain. Ch, cholesterol; FA, fatty acid

Based on the results of our present and previous studies,^13,16^ Fig. 3 illustrates the dynamics and metabolism of VLDL in LCKD-fed mice and presents a working hypothesis of how VLDLR and LPL affect VLDL-triglyceride kinetics. In LCKD-fed wild-type mice, induction of hepatic VLDLR expression and decreased serum LPL activity inhibit the transport of triglycerides by VLDL from the liver to extrahepatic tissues, ultimately promoting steatosis (Fig. 3b). LCKD-induced VLDLR expression promotes triglyceride clearance from VLDL in the liver and the progression of liver steatosis. As LPL activity is necessary for the clearance of triglycerides from VLDL in peripheral tissues,^18^ decreased LPL activity slows triglyceride transport from the liver to peripheral tissues and promotes the development of liver steatosis. In LCKD-fed *ob/ob* mice, the generation of triglyceride-rich VLDL and maintained LPL activity promote the transport of triglycerides from the liver to extrahepatic tissues, ultimately leading to improvement of steatosis (Fig. 3d). Maintained LPL activity promotes the clearance of triglycerides from VLDL in peripheral tissues, which can accelerate triglyceride transport from the liver to peripheral tissues and contribute to the improvement of liver steatosis. LCKD-induced VLDLR expression has a minimal effect on triglyceride-rich VLDL, which is specifically generated in the *ob/ob* strain. LCKD-fed *ob/ob* mice became obese to the same degree as chow-fed *ob/ob* mice, in agreement with the observed effective transport of VLDL-triglycerides from the liver to extrahepatic tissues in these mice.

In *ob/ob* mice, LCKD feeding induces the production of triglyceride-rich VLDL, which has a long plasma residence time due to slow catabolism.^26,27^ This property indicates that triglyceride-rich VLDL is readily delivered to extrahepatic tissues even though VLDLR expression is induced in the liver. As an absence of hepatic leptin signaling leads to elevated triglyceride levels in VLDL particles,^28^ the production of triglyceride-rich VLDL is likely associated with leptin deficiency in *ob/ob* mice.^29^ The maintenance of LPL activity in LCKD-fed *ob/ob* mice could also accelerate the release of triglycerides to extrahepatic tissues. As leptin regulates LPL production and activity in the tissues,^30^ maintenance of LPL activity is also likely associated with leptin deficiency in *ob/ob* mice.^29^

Based on these observations, we conclude that the transport of triglycerides via VLDL from the liver to extrahepatic tissues is inhibited by LCKD-induced hepatic VLDLR expression under conditions of low LPL activity. As this inhibition of triglyceride transport is rescued in leptin-deficient mice, regulating VLDL metabolism by hepatic VLDLR, LPL, and leptin may represent a new therapeutic strategy for preventing diet-induced liver steatosis.

## Methods

### Animals and dietary studies

Dietary studies using female *ob/ob* and wild-type mice (B6.Cg-*Lep*^*ob*^/J and C57BL6J, Charles River Laboratories Japan, Yokohama, Japan) were conducted as reported previously.^13^ CE-2 (CLEA Japan, Tokyo, Japan), composed of 58.2% carbohydrate, 12.6% fat, and 29.2% protein by calories, was used as regular chow. F3666 (Bio-Serv, Frenchtown, NJ) was used as the LCKD, which is composed of 1.7% carbohydrate, 93.9% fat, and 4.4% protein by calories. Five-week-old mice were raised on either regular chow or the LCKD for 7 weeks. During this period, the blood glucose level was monitored at 3:00 p.m. on the first day of every week, and tissue samples were collected at the end of the dietary study. Blood glucose and β-hydroxybutyrate levels were determined using venous blood collected from the tail vein with a Precision Xceed Monitoring System (Abbott Laboratories, Abbott Park, IL, USA).

The Committee for Experiments Involving Animals of the National Institute of Advanced Industrial Science and Technology approved all animal experiments.

### Gene expression analysis

Preparation of total RNA and gene expression analyses were conducted as reported previously.^21^ Agilent Expression Microarray analysis for gene expression profiling in tissues was conducted by Takara Bio (Shiga, Japan). The resulting microarray data were analyzed using the Aqua microarray viewer and Aqua *t* test (Takara Bio) and deposited in the GEO under accession number GSE115342. Relative quantification of target gene expression by real-time PCR was performed using a Light Cycler® 480 II system (Roche, Penzberg, Germany) with the following *Vldlr* gene-specific primers: forward, 5′-gcccgttctactcagtgtatcc-3′; reverse, 5′-gaactcatctgcactacatgttatgtt-3′ (accession number of the *Vldlr* gene: NM_013703, GenBank). Reactions were performed using a KAPA SYBR® FAST qPCR Kit (KAPA Biosystems, Wilmington, MA) according to the manufacturer’s instructions. As it is stably expressed in the liver,^21^ *Eef1a1* was chosen as a housekeeping gene and used as the internal reference for subsequent real-time PCR analyses.

### Immunoblot analysis

Protein extraction and immunoblot analyses were conducted according to a previously reported method^16,31^ using an anti-VLDLR antibody (AF2258; Bio-Techne, Minneapolis, MN, USA).

### Lipoprotein analysis

The triglyceride and cholesterol profiles of serum lipoproteins were determined using a high-sensitivity lipoprotein profiling system employing high-performance liquid chromatography (Skylight Biotech Inc., Akita, Japan), as reported previously.^13^

### Measurement of LPL activity

Serum LPL activity (units ml^−1^) was measured using an LPL Activity Assay Kit (Cell Biolabs, Inc., San Diego, CA, USA) according to the manufacturer’s instructions.

### Analysis of choline-containing phospholipids in the F3666 LCKD and serum

The content of choline-containing phospholipids was determined using a Phosphatidylcholine Assay Kit (Phospholipids C, FUJIFILM Wako Pure Chemical Corp., Osaka, Japan) according to the manufacturer’s instructions.

### Statistical analysis

After determination of variance using the *F* test, statistical significance was evaluated using the two-tailed Student’s *t* test, with statistical significance defined as follows: *P < 0.05, **P < 0.01, and ***P < 0.001.

### Reporting summary

Further information on research design is available in the Nature Research Reporting Summary linked to this article.

## Supplementary information

Supplementary figure S1, S2, S3

Reporting summary checklist

## Data Availability

The datasets generated during and/or analyzed during the current study are available in the GEO repository under accession number GSE115342 or from the corresponding author on reasonable request.
