# Supplementary material for: A low-carbohydrate ketogenic diet induces the expression of very-low-density lipoprotein receptor in liver and affects its associated metabolic abnormalities
Source: NPJ Sci Food. 2019 Dec 2;3:25. doi: 10.1038/s41538-019-0058-4 (PMC6889268; doi:10.1038/s41538-019-0058-4)
Supplement: Supplementary file 1 — Supplementary figure S1, S2, S3 [file 41538_2019_58_MOESM1_ESM.pdf]

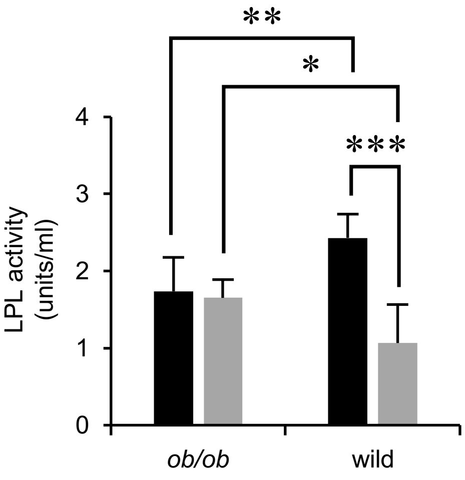

Fig. S1. Effect of LCKD feeding on serum LPL activity.

Serum LPL activity (units ml^{-1}) as measured using an LPL Activity Assay kit (Cell Biolabs, Inc., San Diego, CA, USA). *ob/ob*, n=6-8; wild-type, n=6-7. In all experiments, statistical significance was assessed using the two-tailed Student's *t* test. **P*<0.05, ***P*<0.01, ****P*<0.001, chow vs. LCKD. Black squares and bars, regular chow–fed mice; grey squares and bars, LCKD-fed mice. Mean ± S.D.

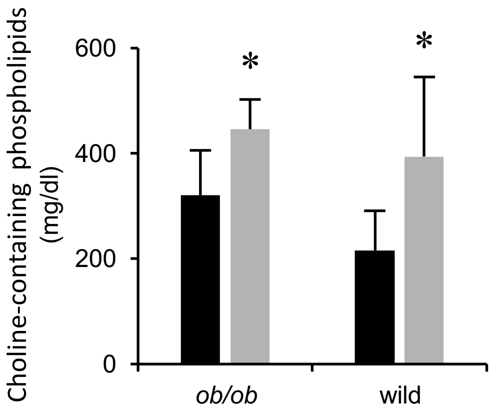

Fig. S2. Serum content of choline-containing phospholipids.

Serum content of choline-containing phospholipids (phosphatidylcholines and sphingomyelins: mg dl^{-1}) as measured using a phosphatidylcholine assay kit (FUJIFILM Wako Pure Chemical Corp., Osaka, Japan). *ob/ob*, n=6-8; wild-type, n=6-7. In all experiments, statistical significance was assessed using the two-tailed Student's *t* test. *P<0.05, chow vs. LCKD. Black squares and bars, regular chow–fed mice; grey squares and bars, LCKD-fed mice. Mean ± S.D.

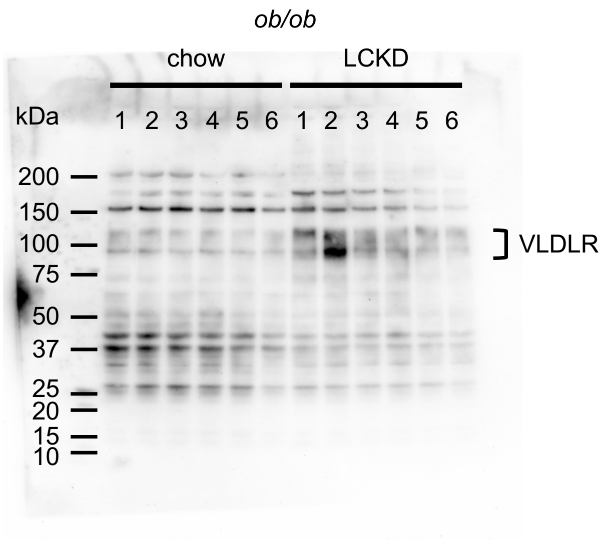

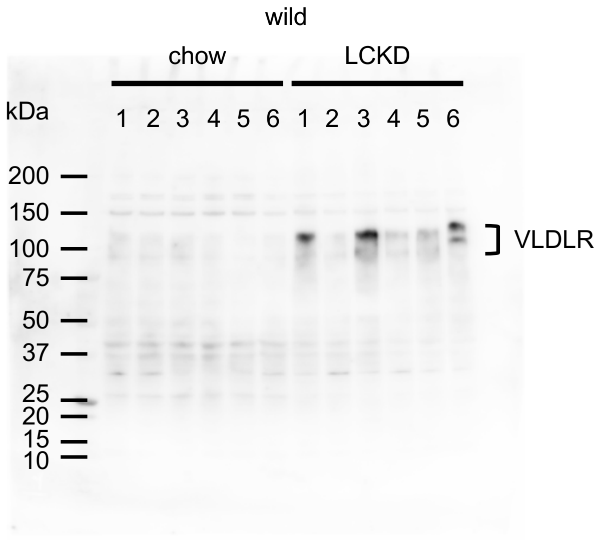

Fig. S3. Full immunoblot images of Fig. 2A and 2B.

Full images of immunoblotting of VLDLR in the liver of individual *ob/ob* (upper panel) or wild-type (lower panel) mice.
